# Type I IFN autoantibodies in three of seven Europeans with life-threatening Usutu virus infection

**DOI:** 10.70962/jhi.20260092

**Published:** 2026-11-03

**Authors:** Adrian Gervais, Paul Bastard, Lucy Bizien, Francesca Trespidi, Micol Angelini, Giada Rossini, Tiziana Lazzarotto, Fausto Baldanti, Francesca Rovida, Alessandro Ferrari, Alessandro Mancon, Davide Mileto, Irene Cassaniti, Francesca Conti, Jonas Schmidt-Chanasit, Dániel Cadar, David M. Florian, Alessandro Borghesi, Anne Puel, Céline Delifer, Pierre Tiberghien, Xavier de Lamballerie, Guillaume André Durand, Judith H. Aberle, Anna Nagy, Yannick Simonin, Aurélie Cobat, Shen-Ying Zhang, Jean-Laurent Casanova

**Affiliations:** 1 Laboratory of Human Genetics of Infectious Diseases, Necker Branch, Institut National de La Santé et de La Recherche Médicale (INSERM) U1163, Necker Hospital for Sick Children, Paris, France; 2 Paris Cité University, Imagine Institute, Paris, France; 3 St. Giles Laboratory of Human Genetics of Infectious Diseases, Rockefeller Branch, Rockefeller University, New York, NY, USA; 4 Pediatric Hematology-Immunology and Rheumatology Unit, Necker Hospital for Sick Children, Assistance Publique-Hôpitaux de Paris, Paris, France; 5 Host-Pathogen Interaction Group and Neonatal Intensive Care Unit, San Matteo Research Hospital (Fondazione IRCCS Policlinico San Matteo), Pavia, Italy; 6 School of Life Sciences, Swiss Federal Institute of Technology, Lausanne, Switzerland; 7 Microbiology Unit, University Hospital of Bologna, Bologna, Italy; 8 Section of Microbiology, Department of Medical and Surgical Sciences, University of Bologna, Bologna, Italy; 9 Microbiology and Virology Unit, San Matteo Research Hospital (Fondazione IRCCS Policlinico San Matteo), Pavia, Italy; 10 Department of Clinical, Surgical, Diagnostic and Pediatric Sciences, University of Pavia, Pavia, Italy; 11 Department of Public Health, Experimental and Forensic Medicine, University of Pavia, Pavia, Italy; 12 Laboratory of Clinical Microbiology, Virology and Bioemergencies, Luigi Sacco Hospital, Milan, Italy; 13 Pediatric Unit, University Hospital of Bologna, Bologna, Italy; 14 Department of Medical and Surgical Sciences, University of Bologna, Bologna, Italy; 15 Department of Arbovirology and Entomology, Bernhard Nocht Institute for Tropical Medicine, Hamburg, Germany; 16 Faculty of Mathematics, Informatics and Natural Sciences, Universität Hamburg, Hamburg, Germany; 17 Virus Metagenomics and Evolution, Bernhard Nocht Institute for Tropical Medicine, Hamburg, Germany; 18 Medical University of Vienna, Center for Virology, Vienna, Austria; 19 Neonatal and Pediatric Intensive Care Unit, Mother, Child and Adolescent Department, Geneva University Hospitals, Geneva, Switzerland; 20 Department of Pediatrics, Gynecology and Obstetrics, University of Geneva, Geneva, Switzerland; 21 Établissement Français du Sang, La Plaine Saint-Denis, France; 22 National Reference Center for Arboviruses, National Institute of Health and Medical Research and French Armed Forces Biomedical Research Institute, Marseille, France; 23 Unité des Virus Émergents, Aix-Marseille University, Corsica University, IRD 190-Inserm 1207-IRBA, Marseille, France; 24 National Center for Public Health and Pharmacy, Budapest, Hungary; 25 Pathogenesis and Control of Chronic and Emerging Infections, University of Montpellier, INSERM, Montpellier, France; 26 Howard Hughes Medical Institute, New York, NY, USA

## Abstract

We previously reported that two of three patients with severe Usutu virus (USUV) disease carried circulating autoantibodies (auto-Abs) neutralizing type I IFNs (AAN-I-IFN). Here, we studied four additional patients, bringing the total number of European patients with life-threatening USUV infection to seven (Austria, *n* = 1; France, *n* = 1; Hungary, *n* = 4; Italy, *n* = 1). Three of these patients (all male, aged 43, 77, and 78 years, one French and two Hungarian) had blood auto-Abs neutralizing at least 10 ng/ml IFN-α2 and IFN-ω and at least 1 ng/ml IFN-β. They suffered from meningitis (*n* = 1), meningoencephalitis (*n* = 1), or myocarditis (*n* = 1). The other four patients were similar demographically and clinically but had no detectable AAN-I-IFN. No AAN-I-IFN were found in the 63 individuals with silent USUV infection tested (P = 6.4 × 10^−4^, Fisher’s exact test). These AAN-I-IFN increased the risk of life-threatening USUV disease by 98.8-fold (95% CI: 4.12–2,369; P = 0.0046, Firth’s bias–corrected logistic regression) and, thus, account for almost half the cases of life-threatening USUV infection in Europe.

## Teaser

We report autoantibodies neutralizing type I IFNs in three of seven European patients with severe Usutu virus infection. The patients are males and aged 43–78 years. These autoantibodies are undetectable in the 63 asymptomatic cases tested.

## Introduction

Usutu virus (USUV) is an emerging mosquito-borne arbovirus of the genus *Orthoflavivirus*, which includes major human pathogens, such as the West Nile virus (WNV), Dengue, and Zika viruses (1, 2). USUV has an enveloped, single-stranded, positive-sense RNA genome of about 11 kb (3). It is transmitted to humans by *Culex* mosquitoes, with various birds serving as a reservoir (4, 5, 6). Humans are incidental, dead-end hosts, typically infected through mosquito bites or, more rarely, organ transplantation (4, 5, 6, 7, 8). Transmission via contaminated bodily fluids (e.g., blood or milk) is theoretically possible, as observed for WNV, but has never been reported for USUV (9, 10). The virus was first isolated from *Culex neavei* mosquitoes in 1959, near the Usutu River in Swaziland, Africa (now Eswatini) (11). It attracted considerable attention following its detection in European birds in Italy (1996) and Austria (2001) (12, 13, 14, 15). It has since become endemic in Italy, Austria, Germany, Hungary, France, Spain, the Netherlands and, more recently, the United Kingdom (4, 16, 17, 18, 19, 20). USUV has never been documented in the Americas, Asia, or Oceania, despite the identification of competent mosquito vectors that could mediate the global spread of the virus in the future (21, 22, 23).

Most human USUV infections are asymptomatic or paucisymptomatic (10, 24). Symptomatic cases typically display nonspecific febrile illness with headache, myalgia, arthralgia, and, occasionally, rash, conjunctivitis, or mild hepatitis (8). However, a subset of patients develop severe neuroinvasive diseases, such as meningitis, encephalitis, meningoencephalitis, and acute flaccid paralysis, sometimes with cranial neuropathies or seizures (6, 16, 20). A recent meta-analysis identified 235 human infections in Europe as of 2023, including 135 with available clinical information; 30% of these cases had symptoms, and the other 70% were asymptomatic. The principal symptom was encephalitis or meningitis, observed in 20%, with no deaths reported (22). The remaining symptomatic cases experienced non-severe symptoms, such as fever and headache. In 2025, over 30 autochthonous cases were reported in humans in France and Italy (25). Three deaths have been documented, all in immunosuppressed individuals (26). Seroprevalence studies indicate broader exposure, with rates of 0.02–3.0% in blood donors and up to 7% in high-risk groups (24, 27, 28, 29). This disparity suggests frequent infection but rare severe disease (∼0.001–0.1% of cases). No vaccine is currently available for humans.

The determinants of severe USUV disease remain incompletely understood (6). Viral genetics may be involved, as human neuroinvasive infections in Europe are disproportionately linked to Europe 2 lineage viruses (30). Epidemiological data indicate that neuroinvasive cases usually occur in patients with acquired immunodeficiency (e.g., solid organ transplant recipients and patients with hematological malignancies or on immunosuppressive treatment) (6, 31). Severe disease can occur in otherwise healthy individuals, but there is currently too little evidence to implicate age or sex as risk factors (6). Studies in mice have shown that the disruption of type I IFN (IFN-I) signaling (as in *Ifnar*^−/−^ or *Stat1*^−/−^ mice) leads to rapid systemic spread of the virus, high viral titers in the central nervous system, and 100% lethality (32, 33, 34). Moreover, studies in vitro and ex vivo have shown that USUV can infect a wide range of human cells, including neurons, astrocytes, microglia, and brain microvascular endothelial cells (30, 33, 35, 36). USUV can evade IFN-I–mediated immunity by inhibiting IFN-β production via disruption of the interaction between MDA5 and MAVS (37). We previously reported that two of three patients with USUV encephalitis that we had studied had autoantibodies (auto-Abs) neutralizing IFN-I (AAN-I-IFN) (38). We hypothesized that other patients with severe USUV infection might also carry AAN-I-IFN.

## Results

We previously investigated a cohort of 34 individuals with USUV infection: 31 individuals with silent infection detected during blood donation and three with severe disease (38). There are no official guidelines for severity classification, but cases were considered severe if they presented with neuroinvasive disease, encephalitis/meningitis, or fatal/disseminated disease. The three patients with severe disease were a 43-year-old Hungarian man (P1), a 78-year-old French man (P2), and an 80-year-old Italian man (P3). P1 and P3 were hospitalized for meningitis and meningoencephalitis, respectively, whereas P2 was hospitalized for myocarditis and systemic inflammatory response syndrome complicated by acute renal failure. P3 died a few days after hospital admission. P1 and P2 had circulating auto-Abs neutralizing high concentrations (10 ng/ml with 1:10 dilutions of plasma or blood) of IFN-α2 and IFN-ω, and intermediate (1 ng/ml) to low (100 pg/ml) concentrations of IFN-β. None of the 31 individuals with silent infection carried detectable amounts of such auto-Abs.

**Figure 1.**
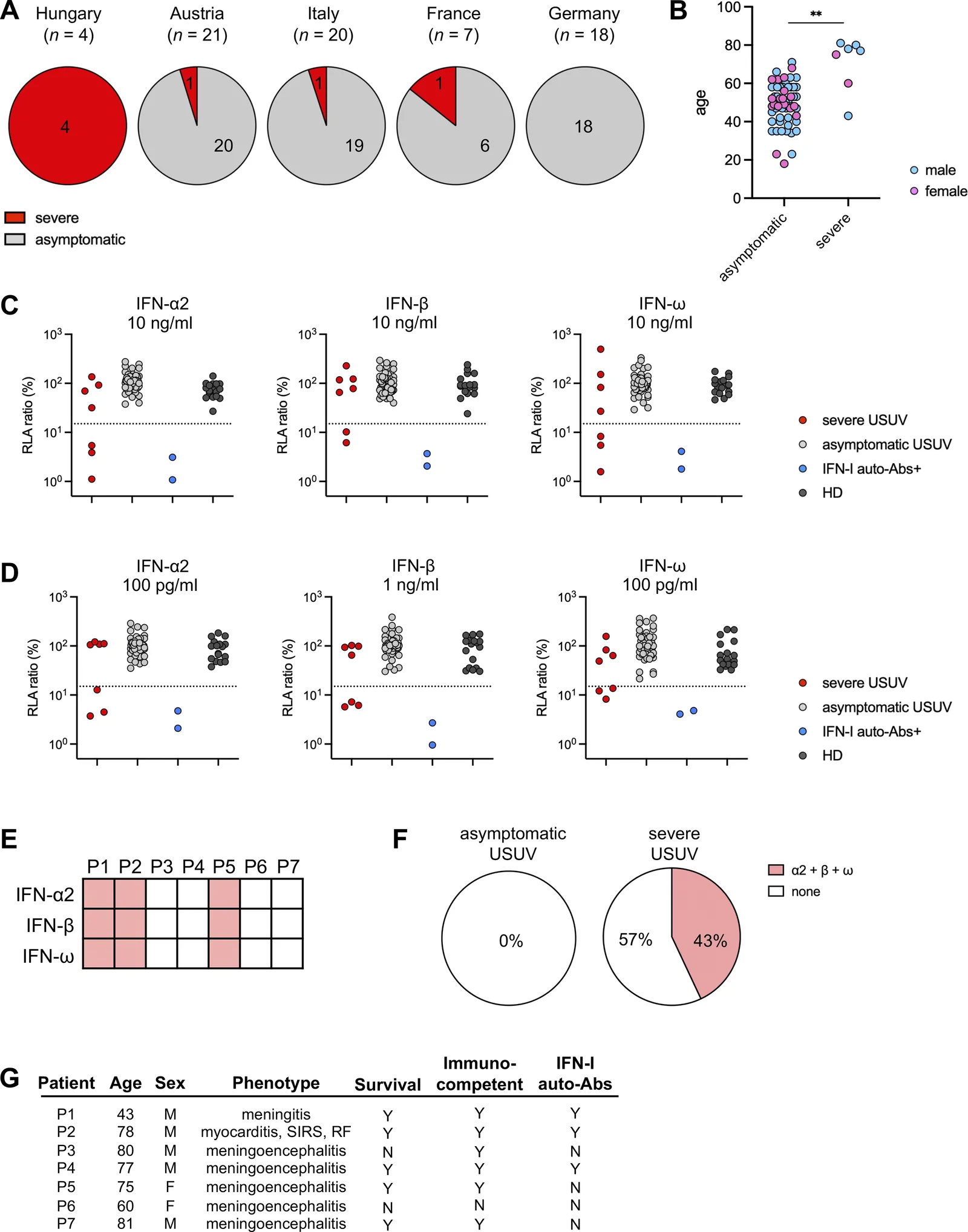
**AAN-I-IFN in patients with severe USUV disease. (A)** 70 USUV-infected individuals from Hungary, Austria, Italy, France, and Germany, and their disease severity. **(B)** Age and sex of the 70 USUV-infected individuals, by severity. Age was compared between the severe and asymptomatic groups by Welch's *t* test. **P < 0.01, Fisher’s exact test. **(C)** Luciferase-based neutralization assay to detect auto-Abs neutralizing 10 ng/ml IFN-α2, IFN-ω, or IFN-β. Samples with a Relative Luciferase Activity (RLA) <15% are considered neutralizing. Each data point represents a single replicate. HD, healthy donors; IFN-I auto-Abs^+^: autoimmune polyendrocrine syndrome type 1 (APS-1) patient. **(D)** Luciferase-based neutralization assay to detect auto-Abs neutralizing 100 pg/ml IFN-α2 or IFN-ω or 1 ng/ml IFN-β. Each data point represents a single replicate. For clarity, serum/plasma and whole-blood samples are not distinguished in this figure. The data showing the distinction between sample types were provided in our previous study (38). HD, healthy donors; IFN-I auto-Abs^+^: APS-1 patient. **(E)** Heatmap depicting the presence of AAN-I-IFN in the seven patients with severe USUV. Red: positive, white: negative. **(F)** Proportion of IFN-I neutralized in USUV-infected asymptomatic individuals versus that in those with severe USUV disease. **(G)** General clinical characteristics of the seven patients with severe USUV disease. Y, yes; N, no; M, male; F, female; SIRS, systemic inflammatory response syndrome; RF, renal failure.

Here, we report findings for four new cases hospitalized for severe USUV disease (P4–P7) and an additional 32 individuals with silent infection. P4 was a 77-year-old Hungarian man with chronic obstructive pulmonary disease, hypertension, diabetes, atrial fibrillation, renal failure, and obesity; P5 was a 75-year-old Hungarian woman with no known comorbid conditions; P6 was a 60-year-old Hungarian woman with pre-existing acute myeloid leukemia leading to severe immunosuppression; and P7 was an 81-year-old Austrian man treated for hypertension with an angiotensin-converting enzyme inhibitor and a β-blocker. He had no recent travel history and was otherwise healthy, leading an active lifestyle, including outdoor sports. He was presumed to be naive to other *Orthoflavivirus* infections and had never been vaccinated against any *Orthoflavivirus* (Fig. 1, A and B). No detailed clinical information was available for the other patients. All four patients were hospitalized for meningoencephalitis. P4, P5, and P7 were immunocompetent and survived the infection, whereas P6, who was severely immunosuppressed, died following infection. Remarkably, one of these four new severe cases, P4, had auto-Abs neutralizing high concentrations (at least 10 ng/ml with 1:10 dilutions of plasma) of IFN-α2, IFN-ω, and IFN-β (Fig. 1, C and D). No such auto-Abs were detected in the serum or plasma of P5, P6, P7, or any of the 32 newly enrolled asymptomatic cases.

Thus, three of seven (43%) severe USUV cases (aged 43–81 years) had AAN-I-IFN able to neutralize high or intermediate concentrations of IFN-I. None of these three patients died following USUV infection. By contrast, no such auto-Abs were detected in the 63 silently infected cases tested from Austria (*n* = 20), Italy (*n* = 19), Germany (*n* = 18), and France (*n* = 6) (P = 6.4 × 10^−4^, Fisher’s exact test) (Fig. 1, E and F). All three patients were male, and all were immunocompetent and not known to suffer from any autoimmune disease. Two of the three presented with central nervous system involvement (Fig. 1 G). We estimated the risk of developing severe USUV disease conferred by AAN-I-IFN, relative to the 63 infected individuals without detectable AAN-I-IFN. USUV-infected individuals with auto-Abs neutralizing both IFN-α2 and IFN-ω at a concentration of 10 ng/ml have a Firth-corrected odds ratio (OR) for developing severe USUV disease of 98.8 (95% confidence interval [CI]: 4.12–2,369; P = 0.0046) relative to those without AAN-I-IFN. This remarkably high OR is consistent with the results presented in our previous studies on the risk conferred by AAN-I-IFN of developing encephalitis following infections with the WNV, tick-borne encephalitis virus (TBEV), chikungunya virus (CHIKV), or vaccination with the live attenuated yellow fever virus or CHIKV vaccines, relative to both infected individuals without AAN-I-IFN and the French general population (38, 39, 40, 41, 42, 43).

## Discussion

These findings confirm and extend our initial report of AAN-I-IFN underlying severe USUV disease (38). Almost half the patients with severe disease studied carried AAN-I-IFN, which were not found in any of the 63 silently infected individuals (P = 6.4 × 10^−4^, Fisher’s exact test). These auto-Abs neutralized IFN-α2, -ω, and -β in all three patients carrying them. No preinfection samples were available, but it is probable that the patients carried AAN-I-IFN before USUV infection, as already reported in patients with other life-threatening viral infections (41, 44, 45, 46, 47, 48), including those triggered by two live-attenuated viral vaccines (39, 49). Both a longitudinal survey of a large Swiss cohort (50) and our more recent study of the B cells specific for IFN-I (51, 52) indicate that the AAN-I-IFN are produced by B cells that have undergone multiple rounds of somatic hypermutation in germinal centers and that once they appear, they persist and diversify. Furthermore, growing numbers of monogenic inborn errors of thymic T cell tolerance are being shown to drive the production of these auto-Abs (53, 54). These auto-Abs are therefore the cause, rather than a consequence, of these severe viral infections, and they account for almost half the European cases of severe USUV disease studied to date. Various autoimmune diseases have been reported in individuals with AAN-I-IFN. It is possible that some of the USUV cases investigated here had such diseases but had not been diagnosed.

We and others have shown that AAN-I-IFN can underlie severe diseases due to four respiratory RNA viruses (SARS-CoV-2 [44, 45, 46, 55, 56, 57, 58, 59, 60, 61, 62, 63, 64, 65, 66, 67, 68, 69, 70, 71, 72, 73, 74, 75, 76, 77, 78, 79, 80, 81, 82, 83, 84, 85, 86, 87, 88, 89], seasonal influenza [90], avian influenza [91, 92], and Middle East respiratory syndrome [MERS] [93]), four predominantly neurotropic orthoflaviviruses (WNV [42, 94, 95, 96], TBEV [40], USUV [38], [this article], Powassan virus [POWV] [38]), two alphaviruses (the Ross River virus [RRV] [38] and CHIKV [43]), two live-attenuated viral vaccines (YFV-17D [49], an *Orthoflavivirus*, and CHIKV-VLA1553 [39], an alphavirus), and two herpesviruses (75, 97, 98) (HSV-1 and HSV-2). The affected organs include the lungs, brain, and liver, depending on the virus concerned. Our results suggest that elderly individuals and those with a history of severe or recurrent viral disease, autoimmunity, or inborn errors of self-tolerance should be screened for AAN-I-IFN before any travel to areas in which these viruses, including USUV, are endemic. Treatment with an IFN-I that is not neutralized by the patient’s auto-Abs may be a relevant therapeutic strategy in patients testing positive for these viral infections before or during hospitalization. Finally, AAN-I-IFN may underlie severe diseases caused by other (arbo)viruses, and genetic deficiencies of IFN-I immunity should be sought in patients with severe diseases caused by these viruses testing negative for AAN-I-IFN.

## Materials and methods

### Patient recruitment and diagnosis of USUV infection

We enrolled an international cohort of individuals with USUV infections. USUV infections were documented by a positive nucleic acid testing result, confirmed either by USUV-specific RT-PCR on blood or urine samples or by USUV neutralization assays on serum or plasma samples, as previously described (99). Written informed consent was obtained from these individuals in their country of residence, in accordance with local regulations and with institutional review board approval. Samples from patients with severe disease were collected between 1 day and 1 wk after the onset of symptoms; samples were collected from the asymptomatic cases at undetermined times after infection, the USUV infection having been discovered fortuitously during blood donation.

### IFN-I neutralization assay

The HEK293T cell–based luciferase reporter assay was performed as previously described (45). These assays were performed on serum or plasma (P2, P4–P7, and silent cases), or whole blood (P1 and P3). Appropriate controls, such as whole blood, serum, and plasma samples from healthy donors and an APS-1 patient with antibodies neutralizing IFN-α2, IFN-ω, and IFN-β, were included to ensure that all these matrices were suitable for the detection of AAN-I-IFN.

### Statistical analysis

Age was compared between patients with life-threatening USUV disease and silently infected individuals by Welch's *t* test. Fisher’s exact test was used to compare the proportion of individuals carrying AAN-I-IFN between patients with severe USUV disease and individuals with silent USUV infection. As no auto-Ab carriers were detected in the silently infected group, the OR was estimated by Firth’s bias–corrected logistic regression with 95% confidence intervals, and the P value was derived by profile-penalized likelihood, as implemented in the logistf package of R software. The OR was calculated only for the combination of auto-Abs neutralizing both IFN-α2 and IFN-ω at 10 ng/ml, as all three auto-Ab–positive patients carried this combination. Given the small sizes of the patient groups, the OR was not adjusted for age or sex.
